# Biogenic synthesis, characterization, and evaluation of synthesized nanoparticles against the pathogenic fungus *Alternaria solani*

**DOI:** 10.3389/fmicb.2023.1159251

**Published:** 2023-04-17

**Authors:** Jeetu Narware, Satyendra P. Singh, Nazia Manzar, Abhijeet Shankar Kashyap

**Affiliations:** ^1^Department of Mycology and Plant Pathology, Institute of Agriculture Sciences, Banaras Hindu University, Varanasi, Uttar Pradesh, India; ^2^Molecular Biology Lab, ICAR-National Bureau of Agriculturally Important Microorganism, Mau, Uttar Pradesh, India

**Keywords:** biogenic nanoparticle, *Alternaria solani*, biocontrol, FTIR-spectroscopy, *Trichoderm* sp.

## Abstract

In the present study, *Trichoderma harzianum* culture filtrate (CF) was used as a reducing and capping agent to synthesize silver nanoparticles (Ag NPs) in a quick, simple, cost-effective, and eco-friendly manner. The effects of different ratios (silver nitrate (AgNO_3_): CF), pH, and incubation time on the synthesis of Ag NPs were also examined. Ultraviolet–visible (UV–Vis) spectra of the synthesized Ag NPs showed a distinct surface plasmon resonance (SPR) peak at 420 nm. Spherical and monodisperse NPs were observed using scanning electron microscopy (SEM). Elemental silver (Ag) was identified in the Ag area peak indicated by energy dispersive x-ray (EDX) spectroscopy. The crystallinity of Ag NPs was confirmed by x-ray diffraction (XRD), and Fourier transform infrared (FTIR) was used to examine the functional groups present in the CF. Dynamic light scattering (DLS) revealed an average size (43.68 nm), which was reported to be stable for 4 months. Atomic force microscopy (AFM) was used to confirm surface morphology. We also investigated the *in vitro* antifungal efficacy of biosynthesized Ag NPs against *Alternaria solani*, which demonstrated a significant inhibitory effect on mycelial growth and spore germination. Additionally, microscopic investigation revealed that Ag NP-treated mycelia exhibited defects and collapsed. Apart from this investigation, Ag NPs were also tested in an epiphytic environment against *A. solani*. Ag NPs were found to be capable of managing early blight disease based on field trial findings. The maximum percentage of early blight disease inhibition by NPs was observed at 40 parts per million (ppm) (60.27%), followed by 20 ppm (58.68%), whereas in the case of the fungicide mancozeb (1,000 ppm), the inhibition was recorded at 61.54%.

## Graphical Abstract

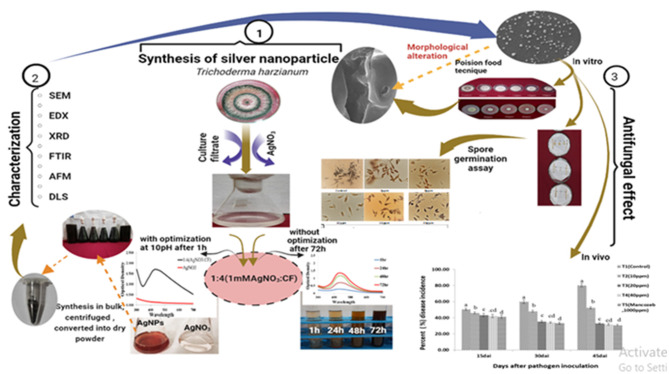

## 1. Introduction

The quantity and quality of agricultural goods continue to deteriorate every year worldwide as a consequence of plant diseases. Plant diseases are a global problem that affects food availability (Rizzo et al., 2021; Kashyap et al., 2022; Manzar et al., 2022a). Early blight is one of the most deleterious diseases of tomato and is caused by *Alternaria solani*. Tomato leaves, stems transplanted at the base, mature plant stems with stem lesions, and fruits with fruit rot are all affected by *A. solani* (Panno et al., 2021). Early blight infection can estimate up to 78% loss in tomato production (El-Ganainy, 2021). *A. solani* performs asexual reproduction *via* multicellular conidia, which can produce apparent necrotic lesions 2–3 days after infection and new conidia 3–5 days later (Rogerson et al., 1987; Chaerani and Voorrips, 2006). Moreover, *A. solani* is a necrotrophic fungus that uses enzymes and a variety of toxins to damage host tissues (Chaerani and Voorrips, 2006) and consumes dead tissues as food and nutrients (Palm, 1997; Chaerani and Voorrips, 2006). It affects all tomato growing regions and causes significant qualitative and quantitative losses at any stage of plant growth, especially in the production of fruits and seeds. The pathogen also causes losses during transport and storage. For up to 0.75–0.77 ton/hectare, estimated yield losses are associated with a 1% increase in disease severity (Saha and Das, 2012). Therefore, crop losses increased with disease severity, and overall crop losses were also observed when disease severity was high. A variety of chemicals, insecticides, and fungicides have been used to remove fungal infestations. These practices can cause health risks and environmental degradation (Özkara et al., 2016; Kumari and John, 2018; Kashyap et al., 2023). Furthermore, the use of agrochemicals may promote the emergence of pathogenic resistance while mitigating the effects of plant diseases (Lamsal et al., 2011; Manzar et al., 2021). Moreover, the consistent use of the same fungicide may increase the risk of the emergence of aggressive fungicide-resistant strains, which may harm beneficial microorganisms that are not intended targets (He et al., 2019; Nottensteiner et al., 2019).

A significant consequence of these effects is that pesticides enter the food chain and leave chemical residues in organisms that are not their actual targets. For all these reasons, suitable alternatives to these phytochemicals as antifungal agents should be explored (Akpinar et al., 2021). The management of microbes, especially phytopathogens, has recently been evaluated using a variety of metallic nanoparticles (NPs) [silver (Ag), zinc (Zn), copper (Cu), titanium (Ti), etc.] synthesized *via* either conventional or unconventional methods (Narware et al., 2019; Chen et al., 2020; Chauhan et al., 2022). Each of these NPs has a specific use as well as unique characteristics. The most frequently synthesized NPs are Ag NPs, and, owing to their extensive and unique range of applications in medicine and agriculture, Ag NPs have attracted the attention of academics as well sparked a global boom of interest among experts in other fields (Guilger-Casagrande and de Lima, 2019). However, by promoting antibacterial action, Ag NPs prevent resistance development (Loo et al., 2018). These characteristics make them safe alternatives to synthetic fungicides in the management of plant diseases. In contrast to chemically synthesized nanomaterials, biological nanomaterials have demonstrated high antibacterial activity against various diseases. Microorganisms have the ability to produce NPs because their cellular machinery has changed to enhance the synthesis of NPs, earning them the moniker “nanofactories” or “nanoparticle producers” (Guilger et al., 2017; Mahawar and Prasanna, 2018). Among microbial agents, fungal bioagents are more suitable than bacteria because they contain a wide variety of intracellular and extracellular proteins and reducing agents (Misra et al., 2022). Under optimal conditions, fungus-based biogenic synthesis offers many advantages in terms of efficiency and production of various metabolites. A further advantage of using fungus as a capping agent for the synthesis of NPs is that fungi naturally produce a large number of antibacterial substances, which have a synergistic effect with metal NPs in the fight against pathogenic microorganisms (Chauhan et al., 2022). The green production of fungi-based NPs is considered an essential branch. Trichoderma strains have a long track record of success. Additionally, recent research findings have shown that these fungi enhance plant resistance, development, and growth, leading to an increase in crop production (Khan et al., 2020; Zin and Badaluddin, 2020). The essential biological component of Trichoderma-mediated mycosynthesis of NPs consists of enzyme reductases, which can function in a bioreductive pathway of biofabricated NP synthesis (Elegbede et al., 2018). Trichoderma is an easy-to-handle fungus and has a number of physiological and technical benefits (Ramírez-Valdespino and Orrantia-Borunda, 2021; Manzar et al., 2022b). A simple, cost-effective, and environmentally favorable approach is to use Trichoderma hyphal extracts, which are beneficial for the production of metal NPs. *Trichoderma harzianum* is a mycoparasitic filamentous fungus that is used in the field for its ability to prevent the occurrence of plant diseases. It contains a number of secondary metabolites and acts as a capping and reducing agent in the production of biologically and environmentally friendly Ag NPs (Zaki et al., 2022). Considering all these qualities of *T. harzianum*, the objectives of the present study were to focus on the synthesis, optimization, characterization, and efficacy of the synthesized Ag NPs against *A. solani*.

## 2. Materials and methods

Silver nitrate (AgNO_3_) was acquired from Central Drug House (P) Ltd. (CDH). The pathogen *A. solani* [Microbial Type Culture Collection and Gene Bank (MTCC) No. 2101] and the biocontrol agent *T. harzianum* (MTCC No. 795) were obtained from the MTCC, CSIR-Institute of Microbial Technology, Chandigarh, India. “Kashi Amrit,” a tomato variety, was obtained from the Indian Institute of Vegetable Research Varanasi. Pathogens and a biocontrol agent were grown in Petri dishes at 27°C in potato dextrose agar (PDA) medium. A field trial experiment was conducted at the Department of Mycology and Plant Pathology of the Institute of Agricultural Sciences, BHU, by creating a containment zone to ensure that there were no adverse or toxic effects on other flora and fauna.

### 2.1. Preparation of culture filtrate

The biocontrol agent was cultured in a potato dextrose broth (PDB) to prepare the filtrate for biosynthetic experiments. For proper development of the mycelium mat, the culture flask was incubated in an orbital shaker at 27°C for 72 h at 150 revolutions per minute (rpm). Mycelium mats were harvested, followed by washing with double distilled water to remove medium components from the biomass. The mycelium mat of *T. harzianum* was incubated (20 g) in 100 ml of sterile, double distilled water for 48 h at 150 rpm. The biomass was filtered using Whatman filter paper no. 1 to obtain the filtrate, which was used as a reducing and capping agent in the synthesis of NPs.

### 2.2. Synthesis of Ag NPs using the culture filtrate

The effects of various factors were investigated according to the instructions of Verma and Mehata (2016). AgNO_3_ was added to the CF to synthesize Ag NPs, and the flasks were incubated in an orbital shaker under dark conditions at room temperature for 72 h. The effects of the concentrations of AgNO_3_ (1 mM) and CF were observed together in different ratios (4:1, 3:2, 2:3, and 1:4). The impact of pH on the reaction mixture was examined at different pHs (8, 9, 10, and 11). The effect of incubation time on both the solution (without altering the pH and CF) and optimal composition reaction mixtures was also recorded [AgNO_3_ 1 Mm:CF (1:4), 10 pH] at 1, 24, 48, and 72 h.

### 2.3. Characterization of the synthesized Ag NPs

The synthesis of Ag NPs was observed by a color change from colorless to dark brown, which was further confirmed by spectrophotometry (Labtronics, model LT-2700 UV). A liquid solution of the synthesized Ag NPs was centrifuged at 12,000 rpm for 10 min, and the precipitate was properly cleaned to remove any undesirable contaminants using sterile distilled water. After purifying and drying the pellets at 60°C, they were analyzed by the Rigaku Miniflex 600 Desktop X-Ray Diffraction System in the scattering range (2θ) of 20°-80°. Their average particle size and size distribution were determined by dynamic light spectroscopy (ZSU5700, Malvern Panalytical, UK). Scanning electron microscopy (SEM) with energy dispersive x-rays (EDX; Carl Zeiss EVO-18) was used for the morphology of the synthesized Ag NPs. Atomic force microscopy (AFM) (NT-MDT, solver nano) was used to confirm the surface morphology and size of the resulting Ag NPs. Fourier transfer infrared (Nicolet iS5 FTIR) was used to investigate the biomolecules involved in the reduction of Ag salts.

### 2.4. Antagonistic effect of biosynthesized Ag NPs against *A. solani*

#### 2.4.1. Growth inhibition of mycelium

The poison food technique was performed using the method described by Falck (1907). Ag NPs were thoroughly mixed with PDA, and their concentration (4, 8, 12, 16, and 20 ppm) was maintained to desired values. The medium was poured into 90-mm Petri plates. A 7-day-old actively growing culture of *A. solani* was carefully cut with the help of the cork borer, aseptically shifted to the center of each Petri plate containing the poisoned medium, and grown in PDA without Ag NPs maintained as control. After 10 days of incubation, the radial growth of the mycelia was measured and the percentage of inhibition over control was calculated using the formula:


$$\begin{array}{l} \;Percentage\;of\;growth\;inhibition\\ =\frac{Mycelial\;growth\;in\;control-Mycelial\;growth\;in\;treatment}{Mycelial\;growth\;in\;control}\times 100. \end{array}$$

On the other hand, the weight of the mycelium was recorded using a method described by Venturini et al. (2002). Different concentrations of Ag NPs (namely, 4, 8, 12, 16, and 20 ppm) were maintained in the PDB. Mycelial discs from young cultures of *A. solani* were inoculated into the medium. The mycelium mat was harvested 10 days after incubation and dried in a tray drier at 60°C, and the dry mycelial weight was recorded.

#### 2.4.2. Spore germination assay

The method was done according to Mohana and Raveesha (2007). A spore suspension was made in sterilized distilled water to assess the efficacy of Ag NPs against spore germination. Different concentrations (4, 8, 12, 16, and 20 ppm) of Ag NPs containing spores of *A. solani* were used for the spore germination assay. Spore suspension of pathogens in distilled water was used as a control. Approximately 100 μl of the mixture solution was placed in a cavity slide and incubated at 28°C ± 1°C in a moist chamber. Three replications were maintained for each treatment, including the control. After 24 h of incubation, slides were examined under different microscopic fields with a ZEISS Axiocam 503 Microscope. Germination percentages were calculated using the formula by Kiraly et al. (1974).

Percent spore germination = (No. of spores germinated)/(Total no. of spores examined) × 100.

#### 2.4.3. Study of morphological alterations

The morphological study was performed by taking the mycelium of the fungal pathogen from Petri plates containing different concentrations of Ag NPs (4, 8, 12, 16, and 20 ppm) used in the poison food technique after 10 days of inoculation. These individual specimens were treated with 0.1 M cacodylate buffer for 1 h after being fixed in 4% glutaraldehyde for 3 h. The specimen was cleaned with distilled water, dehydrated in a graded ethanol series up to 100%, dried to a critical point, and then coated with gold (Ag) using an ion sputter-coater (Quorum) (Lamsal et al., 2011). Samples were examined with a ZEISS EVO-18 research model electron microscope (Germany) at 20 kV. The mycelia were taken from the treated Petri plates, dried to a critical point, and then coated with Ag using an ion sputter-coater (Quorum) (Lamsal et al., 2011).

### 2.5. Field trial study

The field trial was conducted at the Institute of Agricultural Sciences, BHU to study disease incidence (DI) and reduction of *A. solani* in different treatments containing Ag NPs (10, 20, and 40 ppm) and mancozeb (1,000 ppm), leaving the control treatment untreated and seven replicates maintained in each treatment. Plants were inoculated with *A. solani* by foliar application. The prepared Ag NP solution (10, 20, and 40 ppm) and mancozeb (1,000 ppm) were air-sprayed as soon as the first rapid disease symptom appeared on day 15, and the next spraying was scheduled 15 days after the first spray. DI was recorded at different time intervals of 15, 30, and 45 days after pathogen inoculation (dapi) using the 0–9 scale (Ghosh et al., 2009) and was calculated using the formula of Song et al. (2004):


$$\begin{array}{l} \text{DI}\left(\text{\%}\right)=\frac{\text{Σ scale x Numbers of infected plants}}{\text{Highest scale x Total number of plants}}\times 100.\; \end{array}$$

## 3. Results and discussions

### 3.1. Ultraviolet–visible spectroscopy and visual observation and concentration of the CF on the synthesis of Ag NPs

**Figure 1:**
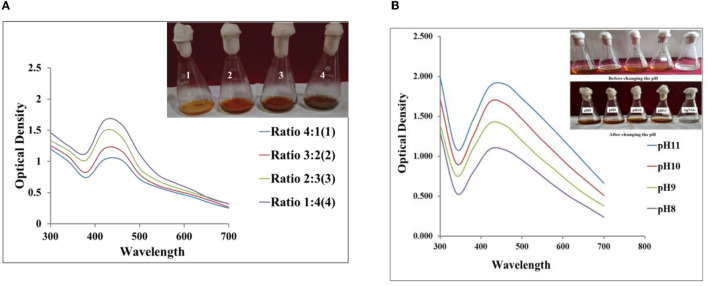
**(A)** Absorption spectra of different ratios of silver nitrate (AgNO_3_; 1 mM) and the culture filtrate (CF) (4:1, 3:2, 2:3, 1:4) at pH 10. **(B)** Absorption spectra of the reaction mixture (AgNO_3_; 1 mM 1:4CF) at different pH values.

Visual examination shows a change in the color of the reaction mixture (AgNO_3_ 1 mM: 4CF) after the addition of AgNO_3_ to the CF. Within 10 min, the color of the reaction mixture began to change to yellowish brown, and after 1 h, it became reddish brown. This shift in color indicates the synthesis of Ag NPs in solution. The collective oscillation of free conducting electrons produced by an interacting electromagnetic field, known as the surface plasmon resonance (SPR) peak, is believed to be the reason for the color shift during the synthesis of metallic NPs (Smitha et al., 2009). Ahluwalia et al. (2014) synthesized Ag NPs using a biocontrol agent and observed color shifts equivalent to the resulting Ag NPs. The synthesis of Ag NPs was further confirmed spectrophotometrically. Figures 1A, B show the color change and ultraviolet (UV) absorption spectrum of the synthesized Ag NPs, peaking at 420 nm. According to Alqadi et al. (2014), peaks at 420 nm are the characteristic feature of Ag NPs (Figures 1A, B).

#### 3.1.1. Effect of pH

The pH of the solution is a significant factor that influences how NPs are synthesized. At different ratios (1:4, 2:3, 3:2, 4:1) of AgNO_3_ (1 mM) and CF concentrations, AgNO_3_:CF (1:4) performs well at pH 10, as shown in Figure 1A. Then, when the pH of the solution (AgNO_3_ 1 mM 1:4CF) increases from 8 to 11, absorption spectra also shifted from 420 to 460 nm (as illustrated in Figure 1B) along with the spectral changes, the intensity of the absorption increases as the pH increases. This confirmed that the reaction mixture of AgNO_3_ 1 mM 1:4CF at pH 10 gave significant results for the synthesis of Ag NPs using *T. harzianum* CF. Additionally, it was shown that pH increased the intensity of the reduction process. A change in the peak wavelength illustrated that, when the pH of the solution rose, particle size also increased. Because the energy needed to excite surface plasmon electrons is small, the absorption maximum shifts toward the longer wavelength area as the particle diameter increases. Previous findings reported that no reaction took place at pH 2.0 because the biomolecules involved in the biosynthesis of Ag NPs were probably downregulated, while highly monodispersed NPs, including those at higher pH (Roopan et al., 2013; Ibrahim, 2015), were produced. This study shows that absorbance increases uniformly as the pH increases from 8 to 11, and it has also been confirmed that alkaline pH is more suitable for the synthesis of Ag NPs. This finding is comparable to a previous study by Vanaja et al. (2013). Alqadi et al. (2014) reported that Ag NPs synthesized at high pH (10 and 11) were more uniform and smaller in size than Ag NPs synthesized at lower pH in the previous study.

#### 3.1.2. Effect of reaction time on the formation of Ag NPs

**Figure 2:**
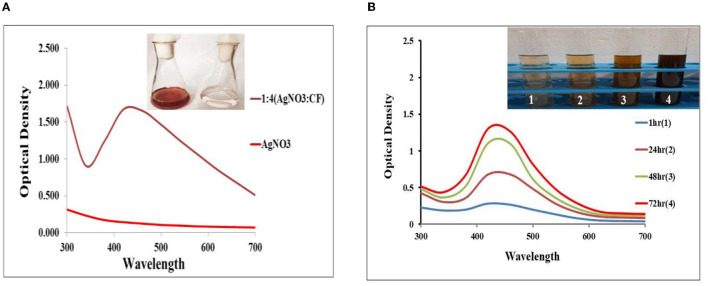
**(A)** Absorption spectra of the synthesized silver nanoparticles (Ag NPs) from AgNO_3_ 1 mM: CF (1:4) at pH 10 after 60 min. **(B)** Absorption spectra at four different reaction times from AgNO_3_ 1 mM: CF (1:4) without a change in pH.

The amount of reddish-brown color was directly linked to the time to incubate the reaction mixture 1:4 (AgNO_3_ 1 mM: CF at pH 10). Within 60 min, the complete color changed, as shown in Figure 2A, and in contrast to the unoptimized CF (Figure 2B), the complete synthesis of Ag NPs took 72 h, after which the color of the reaction mixtures did not change further. Accordingly, it was observed that optimized reaction mixtures fasten the reduction of Ag^+^ to Ag^0^. The synthesized Ag NPs were subsequently analyzed and confirmed by obtaining the corresponding absorption spectrum at 420 nm. The high SPR is responsible for the development of the absorption spectrum. According to Amendola et al. (2010), the SPR band varies with the size and the refractive index of the solution, and the reported absorption band typically depends on the size. Over time, it has been shown that absorbance increases (Figures 2A, B), accelerating the synthesis of Ag NPs. For optimized and unoptimized reaction mixtures, the color of the solution remains constant after 60 min and 72 h, respectively. It has also been confirmed that there are no more Ag salts available for reaction. According to Bhainsa and D'Souza (2006), this rise in absorbance and color intensity might be attributed to the formation of more Ag NPs over time.

### 3.2. SEM and EDX analysis of the synthesized Ag NPs

**Figure 3:**
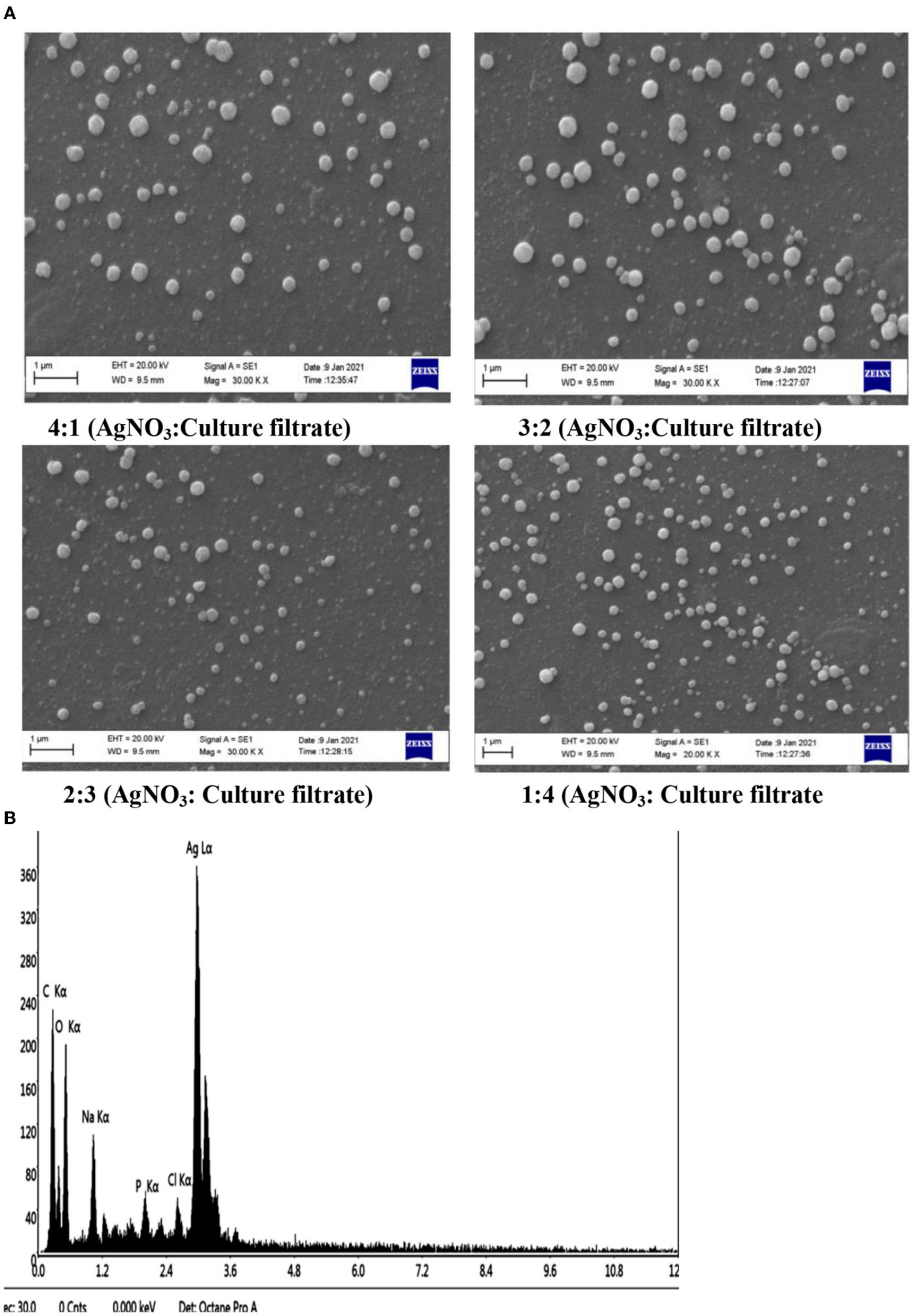
**(A)** Scanning electron microscopy (SEM) micrographs of a variation in the size of the biosynthesized Ag NPs with different ratios of AgNO_3_ and the CF of 4:1, 3:2, 2:3, and 1:4. **(B)** Energy dispersive x-ray (EDX) spectrum of the biosynthesized Ag NPs.

Scanning electron microscopy pictures revealed spherical Ag NPs of various sizes. According to Figure 3A, no direct interaction was found between the two NPs, suggesting that a capping substance has stabilized NPs. Figure 3B shows the results of the NP EDX examination. SEM with an EDX detector was used to evaluate the elemental composition of powder samples. Due to surface plasma resonance, the EDX analysis showed a significant signal at roughly 3 keV in the Ag area (Magudapatty et al., 2001). The spectral signals of carbon, oxygen, sodium, potassium, and chloride were also recorded, indicating that extracellular organic moieties from the CF were adsorbed on NP surfaces. It shows a variation in the size of synthesized Ag NPs with a variation in the different ratios of AgNO_3_ and the CF, which shows that the number of Ag NPs increases and particle size declines in the ratio of AgNO_3_ and the CF (1:4 and 2:3), whereas the reduced number and large particle size were found in the 4:1 and 3:2 ratios of AgNO_3_ and the CF.

### 3.3. Evaluation *via* x-ray diffraction

**Figure 4:**
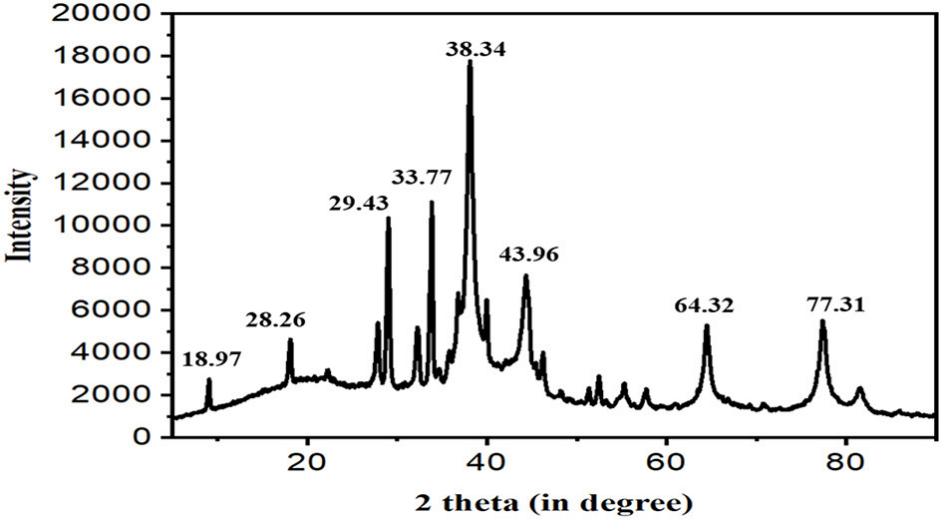
X-ray diffraction (XRD) patterns of the synthesized Ag NPs from MFCF of *T. harzianum*.

The examination of the X-ray diffraction (XRD) patterns, as shown in Figure 4, revealed the crystalline structure of Ag NPs. The diffraction peaks of a face center cubic (fcc) generated from the Ag substrate were indexed as (1 1 1), (2 0 0), (2 2 0), and (3 1 1) reflection planes at 2θ = 38.34, 43.96, 64.32, and 77.31 corresponding lattice plane values (JCPDS card, No. 04-0783) lattice of Ag-3c syn were obtained. The XRD patterns shown here follow the results of previous findings (Das et al., 2013). In addition, peaks appear at 2θ = 33.77, 29.43° lattice plane values (JCPDS card, Nos. 14-0646 and 24-1899) are a lattice of silver (Ag) peroxide and Ag squarate, respectively, were obtained. In addition, 2θ = 28.26 and 18.97 also occurs, probably as a result of organic contaminants in the sample. In other works on Ag NPs, unexplained peaks of XRD were also identifiable in previous studies on Ag NPs (Roopan et al., 2013).

### 3.4. Dynamic light scattering

**Figure 5:**
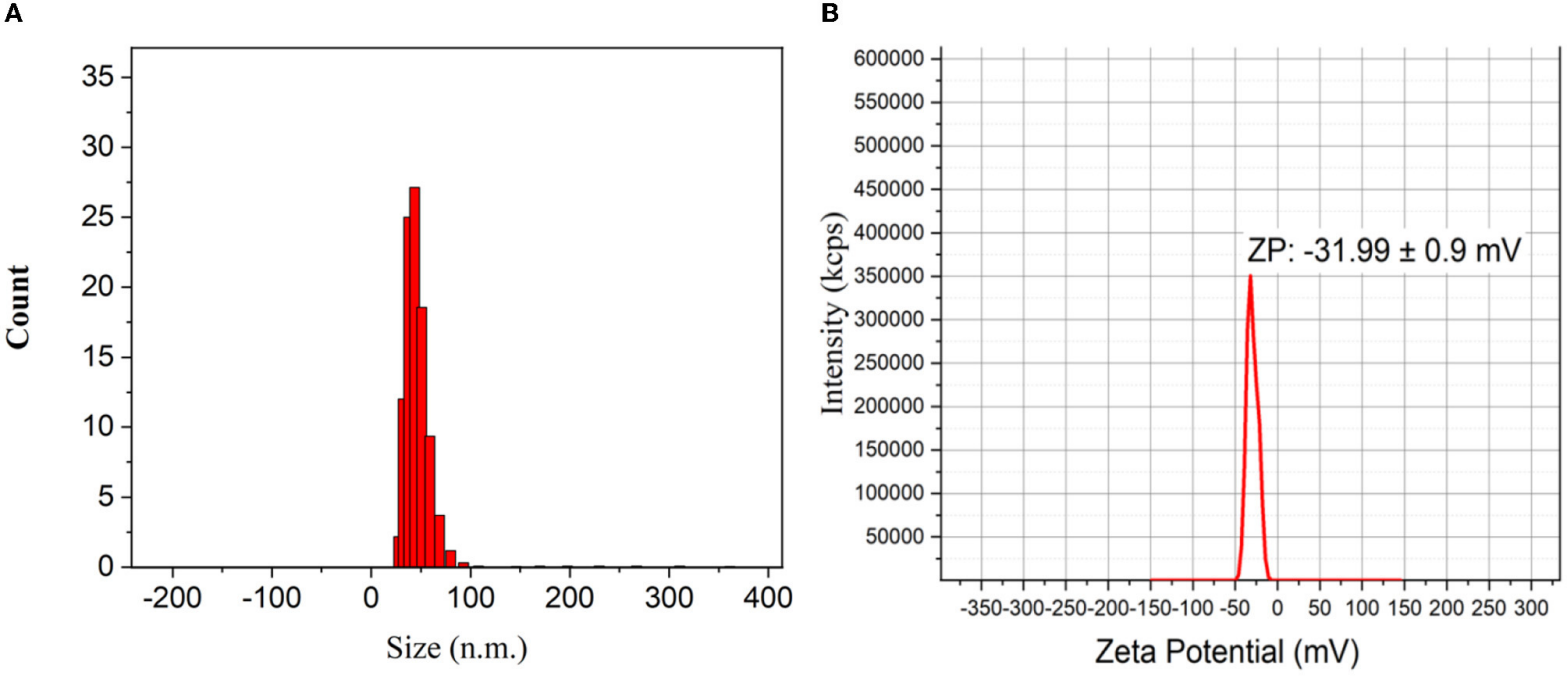
**(A)** Histogram of particle size distribution as obtained from dynamic light scattering (DLS) of Ag NPs. **(B)** Zeta potential of biosynthesized Ag NPs.

The dynamic light scattering (DLS) method was used to measure the particle size and particle size distribution profile of tiny NPs in suspension. This method alters the wavelength of an incoming light by hitting mobile particles with a monochromatic light beam, such as a laser. Particle size is a factor in this transformation. The typical mean particle size of Ag NPs is shown in Figure 5A; NPs have an average size of 43.68 nm. The NP zeta potential was investigated in water as a dispersant. The zeta potential was reported to be −31.99 ± 0.9 mV (Figure 5B). According to Tyagi et al. (2019), Ag NPs repel one another as a result of this negative charge value, reducing their ability to aggregate. This validates the stability of these NPs, showing that there was no clustering of Ag NPs even after 4 months of solution storage.

### 3.5. Atomic force microscopy

**Figure 6:**
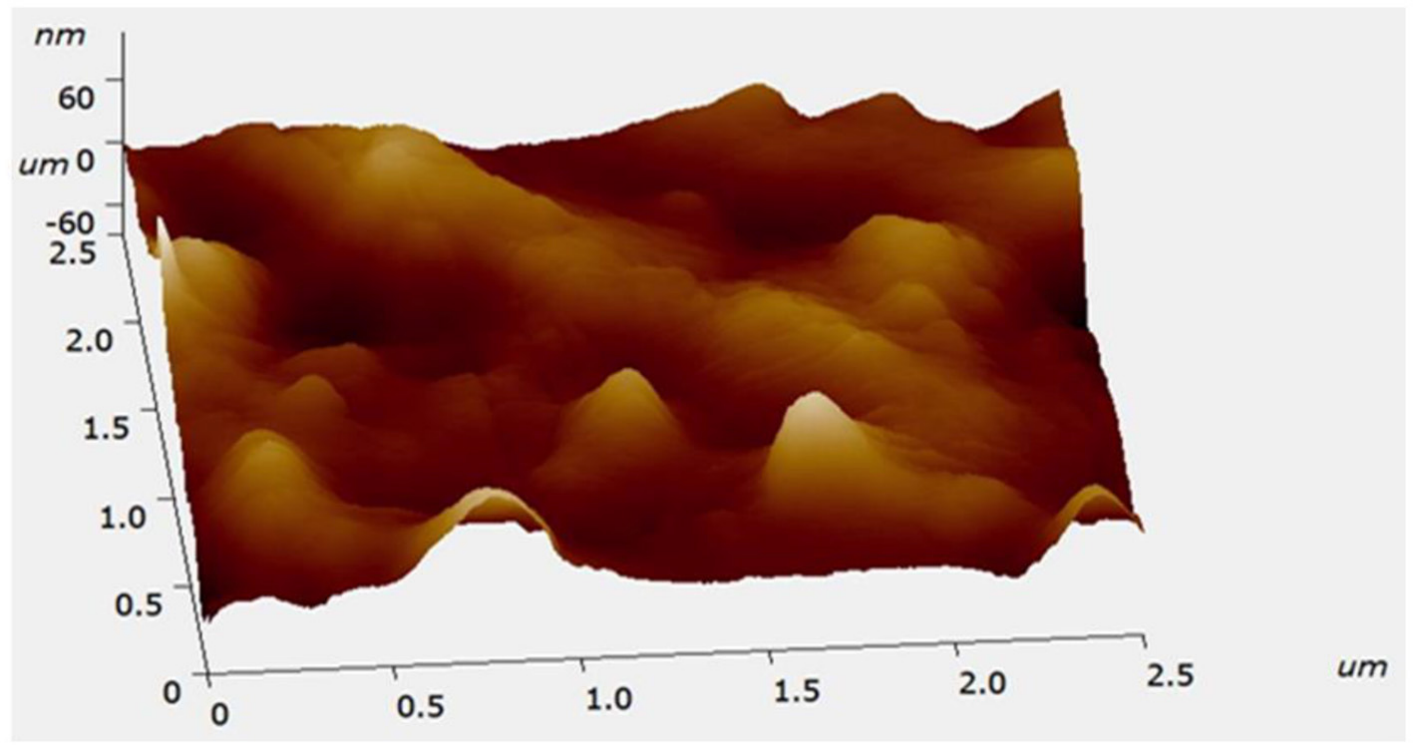
Surface morphology and size of biosynthesized Ag NPs verified by a three-dimensional atomic force microscopy (AFM) image of biosynthesized Ag NPs.

An AFM image of the synthesized Ag NPs is shown in Figure 6. The spherical form of Ag NPs is depicted. The diameter of particles varied from 27.77 to 68.69 nm, and our results were in agreement with earlier reports (Mollick et al., 2012).

#### 3.6. FTIR analysis of the synthesized Ag NPs

**Figure 7:**
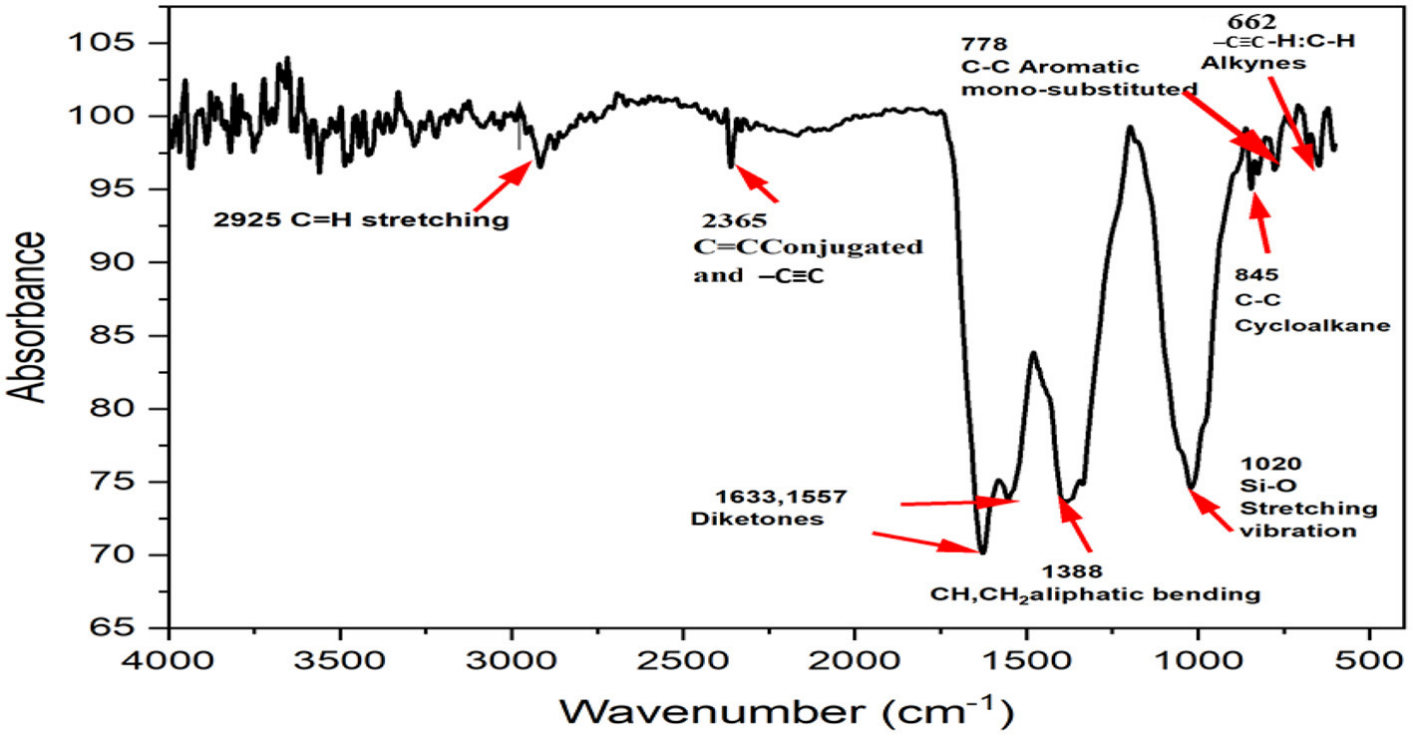
Fourier transform infrared (FTIR) analysis of the synthesized Ag NPs.

Distinct ingredients involved in the reduction and stability of NPs may be identified using FTIR spectroscopy. The Nicolet iS5 FTIR spectrophotometer was used to obtain FTIR spectra for dry and powdered Ag NPs using the attenuated total reflectance (ATR) technique in the range of 4,000–400 cm^−1^ (Figure 7). Ag NPs were blended on KBr pellets and examined using a FTIR spectrometer. Biomolecules specifically bound to the produced Ag NPs were characterized and identified using FTIR spectroscopy. The spectra of the synthesized NPs revealed unique peaks in the range of 2,925, 2,365, 1,633, 1,557, 1,388, 1,020, 845, 778, and 662 cm^−1^ (Table 1). The band between 2,850 and 2,950 cm^−1^ is composed of lipids, proteins, carbohydrates, and phosphines in the aliphatic –CH and –CH_2_ groups between 2,280 and 2,410 cm^−1^ (El Farissi et al., 2021). Some weak bands with triple –C=C and conjugated –C=C bonds between 2,150 and 2,500 cm^−1^ were observed (Jung et al., 2009; Shank et al., 2010). Approximately 1,630–1,725 cm^−1^ C=O of the amide and aromatic ketones, in the range of 1,380–1,385 cm^−1^, 1,020 cm^−1^ shows CH, CH_2_ aliphatic bending and Si–O stretching vibration, respectively, whereas 845, 778, and 662 cm^−1^ represent C–C cycloalkane, C–C aromatic mono-substitution, and –C–H, –C=C alkynes, respectively (Mecozzi et al., 2011). FTIR data revealed that several bioorganic substances from the extract of *T. harzianum* strongly coated or capped NPs. These functional groups are known to bind metal salts to biological materials and facilitate a subsequent reduction to NPs.

**Table 1:** List of band assignments for Fourier transform infrared (FTIR) spectra.

**S. no**.	**Wavenumber (cm^−1^)**	**Functional groups**	**Bond type**
1.	2,850–2,925	Alkane	C–H
2.	2,280–2,410	Phosphine	P–
3.	2,100–2,500		C=C, C=C
4.	1,630–1,725	Amide and Aromatic ketones	C=O
5.	1,380–1,385	Aliphatic bending	CH, CH_2_
6.	1,020	Silica	Si-O
7.	845	Cycloalkane	C–C
8.	778	Aromatic mono-substituted	C–C
9.	662	Alkynes	–C–H,–C=C

#### 3.7. Antagonistic effect of biosynthesized Ag NPs against *A. solani*

##### 3.7.1. Growth inhibition of mycelium

**Figure 8:**
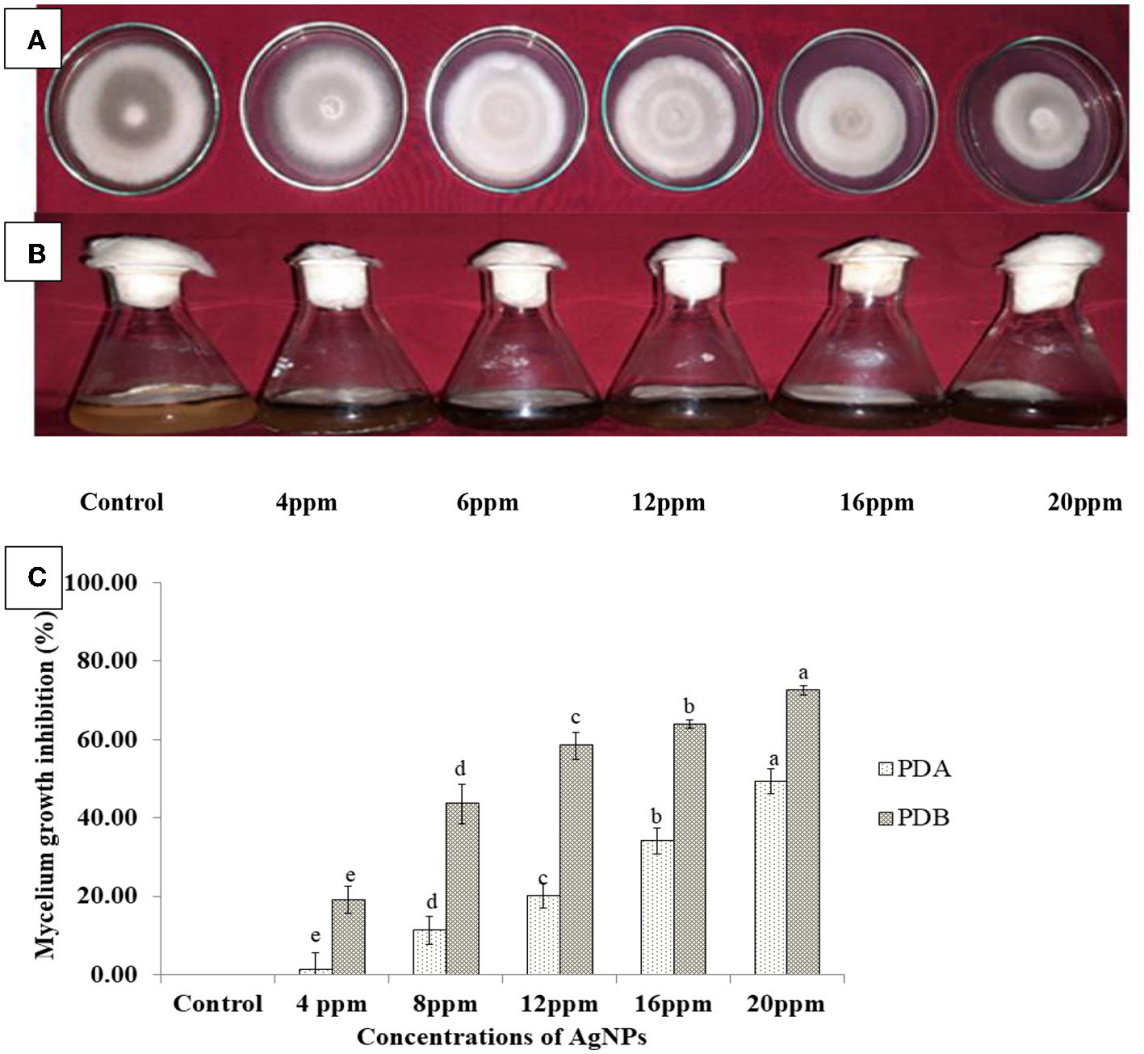
*In vitro* efficacy of the synthesized Ag NPs at different concentrations against *A. solani* on **(A)** potato dextrose agar (PDA) and **(B)** potato dextrose broth (PDB). **(C)** Data were means ± standard errors (SEs) of three replicates, and bars with different letters represent treatments that were significantly different when subjected to Duncan's multiple range test (*p* < 0.05).

Mycelial development of the pathogen *A*. *solani* was inhibited to varying degrees by each of the six treatments of Ag NPs examined at various concentrations. The mean of the results formed the basis of the analysis. A significant reduction in the mycelial proliferation of *A. solani* was observed (Figure 8A) at the concentration of 20 ppm (49.37%) of Ag NPs, followed by 16 (34.18%), 12 (20.25%), 8 (11.39%), and 4 ppm (1.27%), compared with the control (Figure 8C). In addition, inhibition of mycelium (Figure 8B) in PDB was 72.6%, 63.94%, 58.62%, 43.69%, and 19.24% at 20, 16, 12, 8, and 4 ppm, respectively, in contrast to the control (Figure 8C). As a result, all treatments are significantly different from each other. This finding is consistent with a previous study by Kumari et al. (2017), who found a 73.3% reduction in fungal biomass after the 7th day of treatment. The results of this study are consistent with those of previous studies, which have shown that Ag NPs may be employed as antifungal agents to inhibit various fungal plant diseases (Mohanpuria et al., 2007; Wang et al., 2014; Khalil et al., 2019).

##### 3.7.2. Spore germination assay

**Figure 9:**
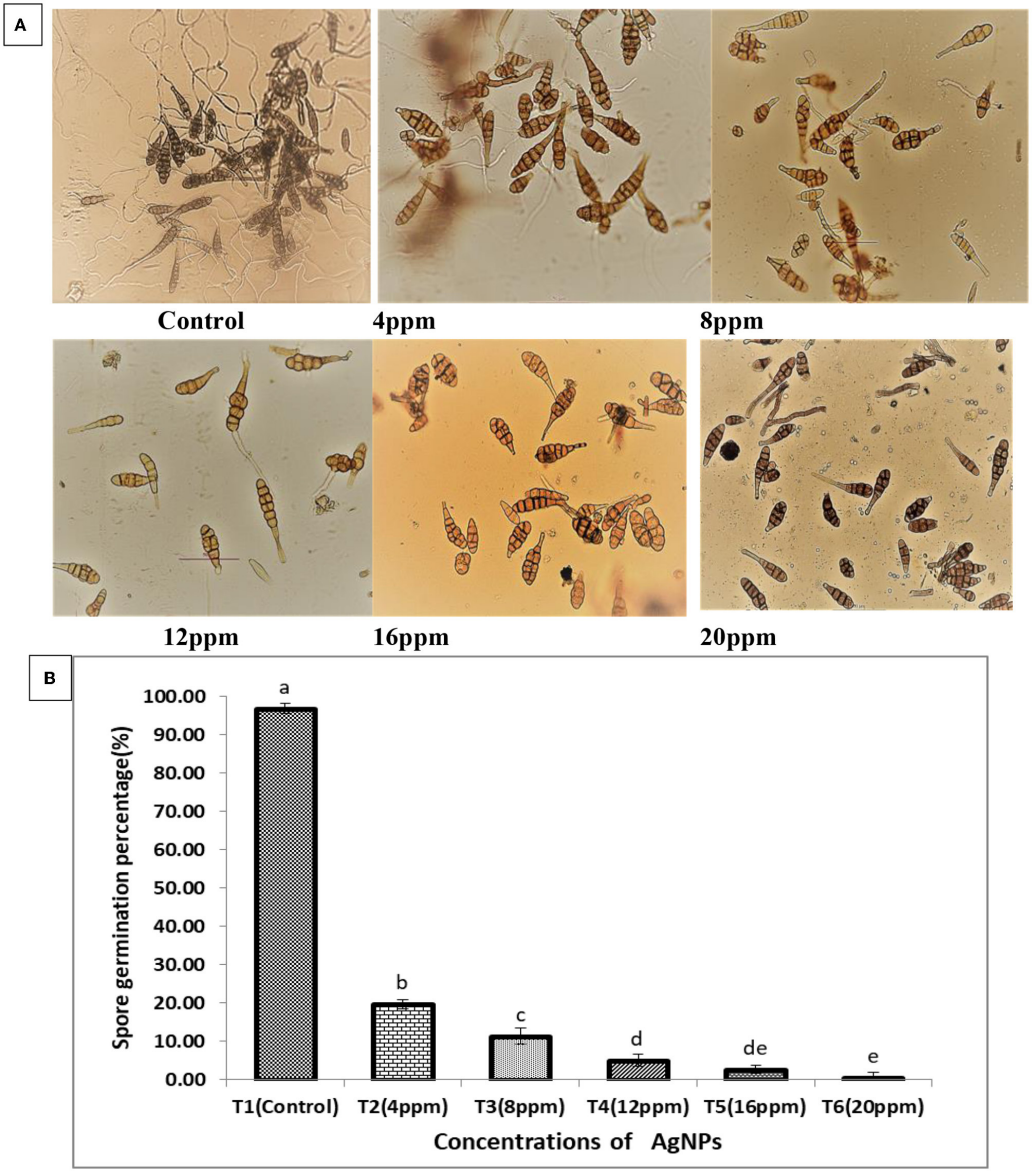
**(A)** Effect of different concentrations of the synthesized Ag NPs on the spore germination rate of *A. solani*. **(B)** The mean of the three replicates of the results are presented as ±SE, and the vertical bars with different letters are significantly different when subjected to Duncan's multiple range test (*p* < 0.05).

Different concentrations of Ag NPs showed a potential inhibitory effect on *A. solani* spore germination. Spore germination was the highest in the control group (96.85%), whereas the percentage of spore germination was significantly lower in different concentrations of Ag NPs than in the control. Germination percentage was as low as 0.65% (20 ppm), 2.58% (16 ppm), 5.08% (12 ppm), 11.30% (8 ppm), and 19.71% (4 ppm) (Figures 9A, B). The result indicated that the lowest concentration of Ag NPs (4 ppm) significantly reduces spore germination. Kriti et al. (2020) reported that the most effective concentration to reduce *Alternaria brassicicola* spore germination rate was 20 ppm, which supported our results. Similarly, the chitosan-Ag NP composite also had an inhibitory effect on the germination of *Colletotrichum gloesporioides* conidia, and the composite with concentrations of 0.1, 1.0, and 10 μg/ml resulted in 44.70% and 78% spore germination inhibition, respectively (Chowdappa et al., 2014). According to the findings, biosynthesized Ag NPs at five distinct concentrations successfully prevented *A. solani* spore germination. The risk of these fungal diseases infecting tomato plants was greatly reduced by preventing spores from germinating.

##### 3.7.3. Study of morphological alterations

**Figure 10:**
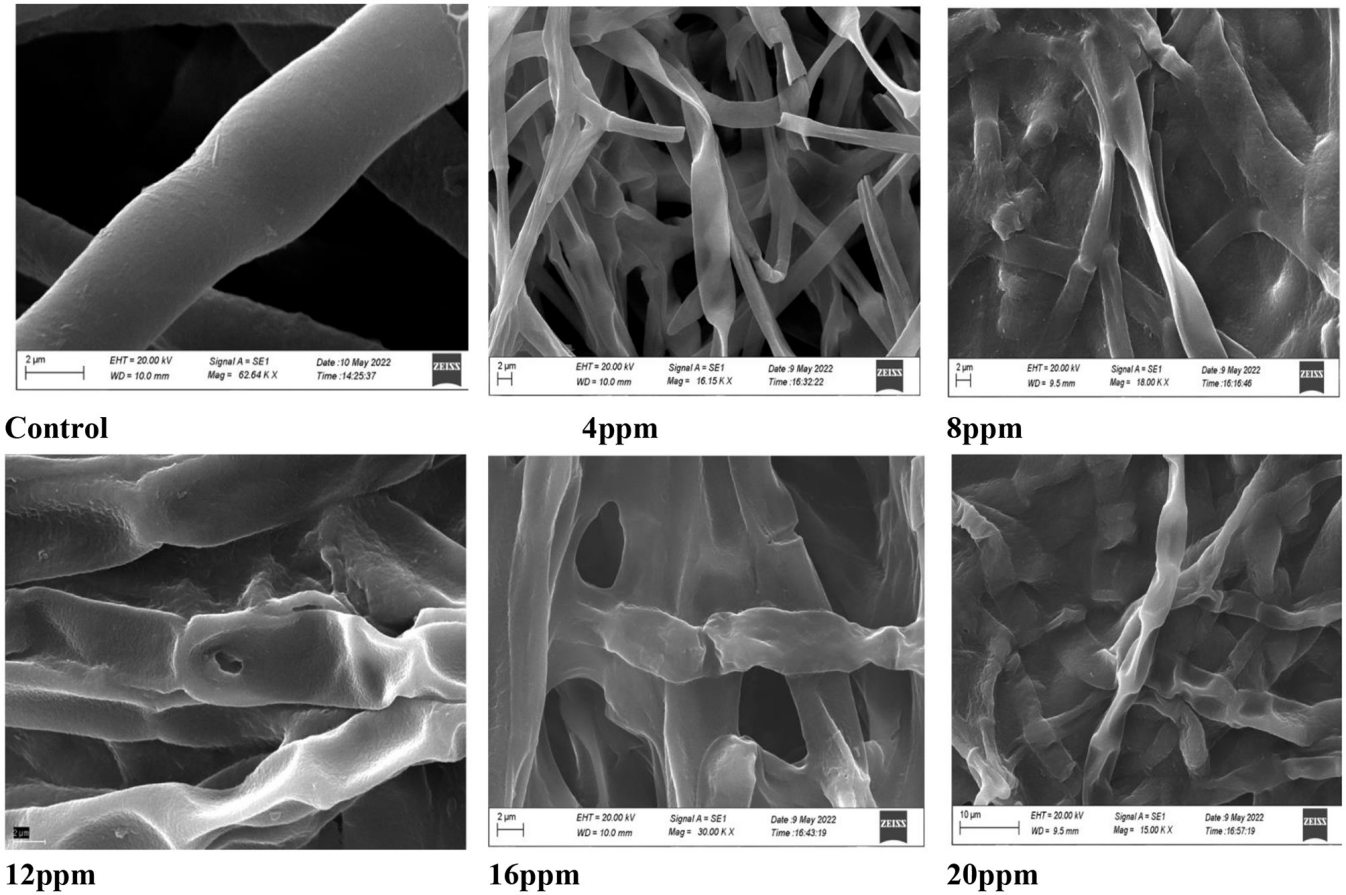
SEM of *A. solani* hyphae treated with different concentrations of silver nanoparticles (Ag NPs) (4, 8, 12, 16, and 20 ppm) with growing fungi but control was without Ag NPs (scale bar = 5 μm).

The mycelial germination of *A. solani* was inhibited by different concentrations of Ag NPs and analyzed by SEM. Microscopic analysis showed that the hyphae were clearly damaged by Ag NPs, but the hyphae not treated with Ag NPs (control) did not seem to be damaged (Figure 10). The hyphae were more severely damaged as the concentration increased. The effect of Ag NPs caused fungal mycelium to settle and become damaged. Three days after exposure to different concentrations of Ag NPs, fungal hyphae were studied and showed abnormalities in hyphal wall morphology and mycelial development. In injured hyphae, the hyphal wall layers were also broken and many hyphae ruptured. This result was correlated with previous findings (Al-Othman et al., 2014; Xia et al., 2016), which confirmed that Ag NPs affected cellular processes of the fungus and caused distortion and shrinkage of hyphae due to the surface damage to the mycelium resulting in intracellular components. When Ag ions build up quickly due to ion efflux dysfunction, they can disrupt cellular functions at low concentrations, including metabolism and respiration by interacting with molecules. Additionally, Ag ions are known to generate reactive oxygen species when they interact with oxygen. These reactive oxygen species are harmful to cells because they harm proteins, lipids, and nucleic acids (Hwang et al., 2008). In accordance with the findings of this study, earlier research has shown that the accumulation of Ag NPs on the cell membrane may result in damage to the cell membrane, which leads to the leakage and release of cellular contents like deoxyribonucleic acid (DNA) and proteins (Ghosh et al., 2009; Dizaj et al., 2014; Fouad et al., 2017; Bruna et al., 2021).

#### 3.8. Field trial study

**Figure 11:**
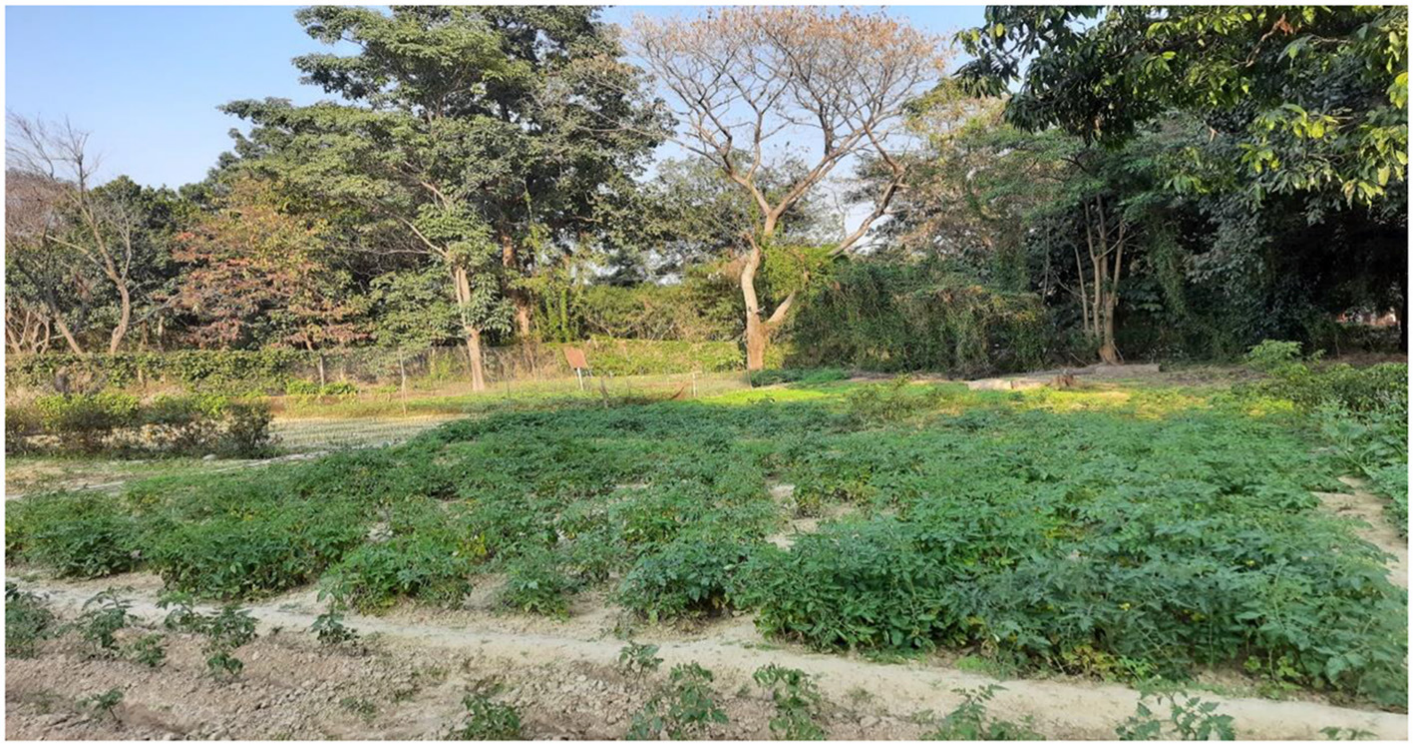
Field study experimental plot at the Institute of Agricultural Sciences, BHU, Varanasi.

**Figure 12:**
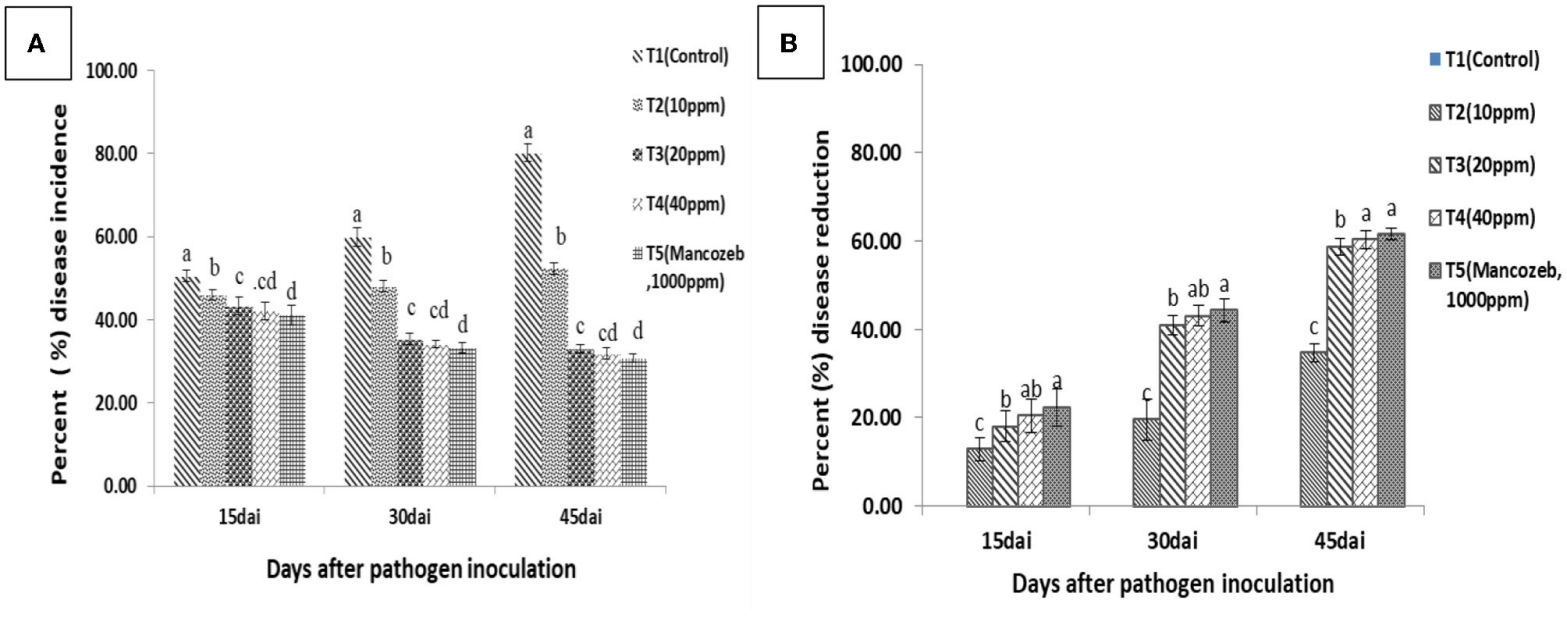
**(A)** Disease incidence (DI) and **(B)** disease reduction in Ag NP-treated tomato plants against early blight of tomato in the field. The results were obtained 15, 30, and 45 days after pathogen inoculation (dapi). Commercial fungicide mancozeb (1,000 ppm) was used as a positive control. Error bars in the figure represent standard deviation (SD) of means. Distinct superscript letters represent data significantly different from other treatments (*p* ≤ 0.05; Duncan's multiple range test).

**Figure 13:**
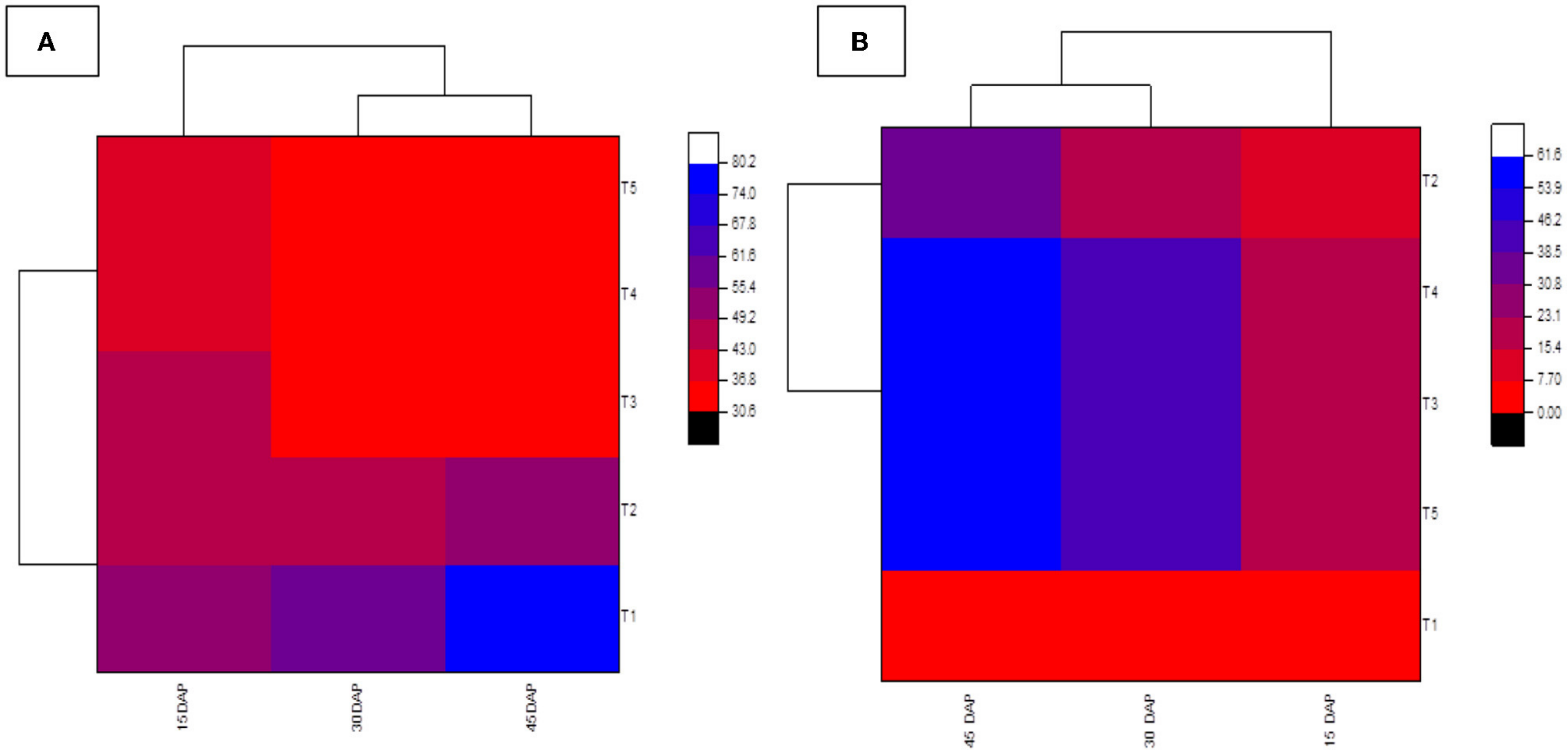
Heatmap showing **(A)** disease incidence and **(B)** disease reduction in Ag NP-treated tomato plants against early blight of tomato in the field. The results were obtained at 15, 30, and 45 dapi. A commercial fungicide, mancozeb (1,000 ppm), was used as a positive control. Ag NP treatments were T1 (control), T2 (10 ppm), T3 (20 ppm), T4 (40 ppm), and T5 (Mancozeb).

Data recorded from the field study (Figure 11) clearly showed that biologically synthesized Ag NPs significantly reduced the percentage of DI compared to untreated controls (80.10%). The minimum PDI was recorded for chemical (mancozeb) treatment (30.79%), and 31.79%, 33.06%, and 52.32% of mancozeb were recorded at 40, 20, and 10 ppm in the case of Ag NP treatments, respectively. These results clearly indicated that the PDI of Ag NP treatments was significantly lower than that of untreated controls. It is also clear from Figure 12A that PDI is non-significant in treatments T3 (20 ppm) and T4 (40 ppm). T5 (61.54%) treatment with mancozeb recorded the maximum percentage of disease reduction (PDR), followed by T4 (60.26%), T3 (58.67%), and T2 (34.64%), i.e., 40, 20, and 10 ppm, respectively (Figures 12B, 13). Lamsal et al. (2011) found a 9.7% reduction in DI when Ag NPs (50 ppm) were applied before the outbreak of pepper anthracnose under field conditions. In a study by Das et al. (2022), the highest percentage of purple blotch disease inhibition was 100 ppm (80.93%), followed by 75 ppm (77.17%) under field conditions. According to many studies, Ag NPs bind to the cell surface, inhibit cell permeability and respiratory functions, and may engage with the membrane surface and enter the organism simultaneously (El-Kassas and Ghobrial, 2017; Quiroz-Hernandez et al., 2020). The results also showed that the concentration and size of Ag NPs determine the efficacy of the inhibition and reduce the maximum DI of *A. solani* at a lower concentration of Ag NPs (20 ppm) when compared to the commercial fungicide mancozeb (1,000 ppm), which may be due to the smaller particle size and larger surface area of Ag NPs.

## 4. Conclusion

In this study, the straightforward, one-pot microbial synthesis of stable Ag NPs using *T. harzianum* CF at room temperature was performed. In terms of reaction time and stability of the produced NPs without external stabilizers or reducing agents, the process of synthesis was considered effective. The pH, culture concentration, and reaction duration are all crucial factors in the synthesis of Ag NPs. By changing these factors, the size and form of the NPs produced can be changed. The synthesis of Ag NPs is enhanced by time and high concentrations of CF and alkaline pH. Ag NPs synthesized using *T. harzianum* CF are characterized by crystallinity, uniformity, sphericity, and monodispersity with an average particle size of 43.68 nm. The synthesized NPs remained stable for 4 months, according to DLS. *T. harzianum* CF turns out to be an environmentally friendly and quick method for synthesis, offering a cheap and effective technique to create Ag NPs. The synthesized Ag NPs demonstrated an effective antifungal action against the target pathogen. In comparison to physical and chemical procedures, this microbiological synthesis approach appears to be economical, non-toxic, and ecologically sound. It would be appropriate for creating a biological process for large-scale manufacturing. It is feasible that a deterioration in membrane integrity led to the antifungal activity observed in this study. The findings from the field investigation showed that the use of the synthesized Ag NPs against *A. solani*, the organism that causes early blight on tomatoes, significantly reduced DI.

## Data availability statement

The original contributions presented in the study are included in the article/supplementary material, further inquiries can be directed to the corresponding authors.

## Author contributions

JN conceived the idea of the manuscript, performed experiments and inputs for each specific section, and drafted the manuscript. SS, NM, and AK edited, compiled, and finalized the final draft. All authors have read and agreed to submit current version of the manuscript.
